# Dispersion and Aggregation Fate of Individual and Co-Existing Metal Nanoparticles under Environmental Aqueous Suspension Conditions

**DOI:** 10.3390/ma15196733

**Published:** 2022-09-28

**Authors:** Jejal Reddy Bathi, Shuvashish Roy, Syed Tareq, Gretchen E. Potts, Soubantika Palchoudhury, Samantha O. Sweck, Venkataramana Gadhamshetty

**Affiliations:** 1 Civil and Chemical Engineering, University of Tennessee at Chattanooga, 615 McCallie Ave, Chattanooga, TN 37403, USA; 2 Chemistry and Physics, University of Tennessee at Chattanooga, 615 McCallie Ave, Chattanooga, TN 37403, USA; 3 Chemical and Materials Engineering, University of Dayton, 300 College Park Ave, Dayton, OH 45469, USA; 4 Civil, and Environmental Engineering, South Dakota School of Mines and Technology, 501 E. St Joseph Street, Rapid City, SD 57701, USA; 5 2-Dimensional Materials for Biofilm Engineering Science and Technology (2DBEST) Center, South Dakota School of Mines and Technology, 501 E. St. Joseph Street, Rapid City, SD 57701, USA

**Keywords:** metal nanoparticles, dispersion, aggregation, nanoparticle analysis, environmental fate

## Abstract

The use of diverse metal nanoparticles (MNPs) in a wide range of commercial products has led to their co-existence in the aqueous environment. The current study explores the dispersion and aggregation fate of five prominent MNPs (silver, copper, iron, nickel, and titanium), in both their individual and co-existing forms. We address a knowledge gap regarding their environmental fate under turbulent condition akin to flowing rivers. We present tandem analytical techniques based on dynamic light scattering, ultraviolet-visible spectroscopy, and inductively coupled plasma atomic emission spectroscopy for discerning their dispersion behavior under residence times of turbulence, ranging from 0.25 to 4 h. The MNPs displayed a multimodal trend for dispersion and aggregation behavior with suspension time in aqueous samples. The extent of dispersion was variable and depended upon intrinsic properties of MNPs. However, the co-existing MNPs displayed a dominant hetero-aggregation effect, independent of the residence times. Further research with use of real-world environmental samples can provide additional insights on the effects of sample chemistry on MNPs fate.

## 1. Introduction

The global market for metal nanoparticles (MNPs) is expected to reach as high as USD 25 billion by 2022 due to their versatile applications [1]. These MNPs will gradually enter the natural environment, including surface water (e.g., rivers, lakes) [2,3]. Dispersion and subsequent fate and transport of MNPs in the aquatic environment are influenced by both physicochemical properties and water chemistry parameters [4,5]. Their small size and large surface area, makes MNPs agglomerate and not disperse in water easily [6]. The agglomeration effects are complex and become unpredictable when they interact and co-exist, especially under turbulent environmental conditions, such as in rivers [7,8,9]. For example, the interplay between ZnO and Zn with the co-existing TiO_2_ nanoparticles (NPs) has been reported to control the levels of Zn ions in aqueous media [10].

Turbulent conditions promote collision frequency, even when they exist in very small concentrations (e.g., ng/L), resulting in aggregation and disaggregation of MNPs [11]. Mixing lengths (time for complete mixing of the introduced material) in small rivers (flow rate (Q) ~ 10 m^3^/s) occur at a scale of few meters (few minutes), while in large rivers (Q > 1000 m^3^/s) such mixing may happen over thousands of kilometers (days), and they depend on the hydraulic and geomorphological conditions of rivers [12]. The dispersion of MNPs under turbulent conditions can be impacted at both molecular scales (diffusion and chemical transformation) and macro scales (advection and turbulence) [13,14]. The net dispersion behavior cannot be predicted with advection-dispersion models based on Fickian diffusion theory alone, especially when MNPs display a non-conservative behavior [14]. Hence, laboratory experiments can aid in calibration of computer models to study MNPs fate in complex real-world conditions.

Here, we address a knowledge gap regarding the impacts of mixing induced by turbulence conditions (similar to river systems) on the dispersion and aggregation behavior of prominent co-existing MNPs on a temporal scale. We used mechanical sonication for simulating turbulent mixing conditions observed in small rivers with residence times of 0.25 h to 4 h [15]. We used ultrasonic bath sonication for mixing, as probe sonication has been reported to alter the media properties, including increase in media temperature [16]. We note that the sonication may not be a total true representation of a river but is useful to approximate the conditions. We applied tandem analytical techniques to fully quantify and mechanistically understand the dispersion of both the individual and co-existing forms of MNPs. Specifically, we studied silver (Ag), copper (Cu), iron (Fe), nickel (Ni), and titanium (Ti) as technologically relevant MNPs, owing to their prominent use and reported detection in environmental systems [5].

Another important knowledge gap related to MNPs in the environment is accurate characterization of their toxicity. Although recent green synthesis approaches gain momentum for minimizing toxicity associated with MNPs production, there exists a toxicity threat to the environment from MNPs, requiring characterization of MNPs toxicity [17,18,19,20]. However, a central problem in characterizing MNPs’ fate and toxicity is analytical limitation techniques for their characterization at low concentration [17], which can be overcome by tandem analytical techniques for measuring the same characteristic so that the weight of evidence of quantified characteristics can be improved [21]. Though advanced tools, such as single particle-inductively coupled plasma-mass spectrometry, can yield detailed characterization of the MNPs, their use is limited for wide-scale application due to high costs and limited access. Inductively coupled plasma-atomic emission spectroscopy (ICP-AES) is less costly and commonly available in many environmental labs. ICP-AES can determine the individual elemental concentration, regardless of size or speciation [22]. In this study, a tandem technique approach containing ICP-AES combined with dynamic light scattering (DLS) and ultraviolet-visible (UV-Vis) absorption spectroscopy to achieve an inexpensive approach for reliably characterizing the MNPs in aqueous samples is demonstrated. More details on this analytical approach can be found in our earlier publication in this journal [21], hence the research presented in this paper is an extension of our earlier published research.

## 2. Materials and Methods

*Sample Preparation and Suspension Settings*: Non-coated spherical MNPs (Ag, Cu, Fe, Ni, and Ti) with properties noted in Table 1 were obtained from Sky Spring Nanomaterials, Inc., Houston, TX, USA. Aqueous samples were prepared by diluting weighted amounts of MNPs in 50 mL of Millipore water in a glass beaker. The initial pH of the sample was measured to be 6 and no further adjustment to the sample pH was made. Since suspension time and concentration of MNPs in real environments is variable, suspension times (0.25–4 h) and MNP concentrations were selected to match the conditions in small rivers (Q = 10 m^3^/s) near the vicinity of wastewater discharges (Table 1). Co-existing samples were prepared with each MNP concentration of 12 ppm. The samples were subjected to ultrasonication for 0.25, 0.5, 1, 2, and 4 h using a bath sonicator rated at 40 kHz, 115 V (Branson 1800, Fisher Scientific, Branson, MO, USA). At the end of each time interval, samples were drawn from the beakers using a glass pipette for suspension characterization and MNPs quantification.

*Suspension Characterization*: The extent of MNPs dispersion in the solutions at each suspension time was characterized using UV-Vis (Spectronic 200 Ultraviolet-Visible Spectrophotometer, Thermo Scientific) over the wavelength range of 340–1000 nm. The UV-Vis spectra were collected for each suspension time using VISIONlite5 software (Fisher Scientific, USA) and plotted using the Kaleidagraph graphical software. The absorbance results were reported as an average of three measurements.

The hydrodynamic size, zeta potential (ζ-potential), and polydispersity index (PdI) at each suspension time were investigated at room temperature on a Litesizer^TM^ 500 Particle Analyzer (Anton Paar, Ashland, VA, USA) DLS, equipped with ζ-potential capability. Kalliope^TM^ software was used for data acquisition. Hydrodynamic size, in three metrics (e.g., intensity-weighted size, volume-weighted size, and number-weighted size), of each MNPs dispersion sample were reported as an average of three measurements. For all analyses using DLS, the suspension times of 0.25, 2, and 4 h were chosen as representative conditions for this study. For ζ-potential, three consecutive measurements were conducted for each sample and the average value was used as reliable representation of the sample ζ-potential. The instrument’s automatic measurement angle, general analysis model, and advanced cumulant model were selected as other input parameters for each measurement.

*MNPs Quantification*: The concentration of MNPs in the dispersion samples at each suspension time was measured using ICP-AES. Before ICP-AES analysis, the MNPs solutions were filtered using 0.22 µm polyethersulfone filter tips (Environmental Express, Charleston, SC, USA), with 10-mL Luer-lock syringes (Fisher Scientific Inc., Pittsburgh, PA, USA) to remove any large particles that could clog the nebulizer. The filter size was such that it would allow the dispersed MNPs to pass through for further analysis by ICP-AES [23]. The operating conditions of the ICP-AES, calibration equations for the elemental MNPs, correlation coefficients, calculated limit of detection (LOD), and limit of quantitation (LOQ) are provided in the Supplementary Materials. All the calibration curves have a 10^4^ linear dynamic range with excellent fit, indicated by correlation coefficients greater than 0.999. For instrument calibration, single element standards (Spex Certiprep, Metuchen, NJ, USA, 1000 ppm, 2% nitric acid) were obtained from Fisher Scientific Inc. The multi-element ICP-AES standard solutions (Ag, Cu, Fe, Ni, Ti) were prepared by mixing and diluting the single element standards to 0.01, 0.1, 1.00, and 10.0 mg/L in Millipore water (18 MΩ·cm), resulting in less than 0.02% nitric acid solution.

## 3. Results and Discussion

### 3.1. Suspension Time versus Dispersion Quality

A large absorbance over the entire wavelength range in UV-Vis typically indicates a higher quantity of NPs dispersed or suspended in the solution. Literature reports that the extinction coefficient and hence absorbance of Ag NPs increases with particle diameter (d) following a power function (i.e., d^0.77^) [24]. Therefore, increased agglomeration can lead to increased absorbance in the case of Ag NPs. We correlate our UV-Vis measurements with DLS size results for the individual and co-existing MNP samples to understand the influence of mixing on the dispersion of the MNPs.

**Figure 1:**
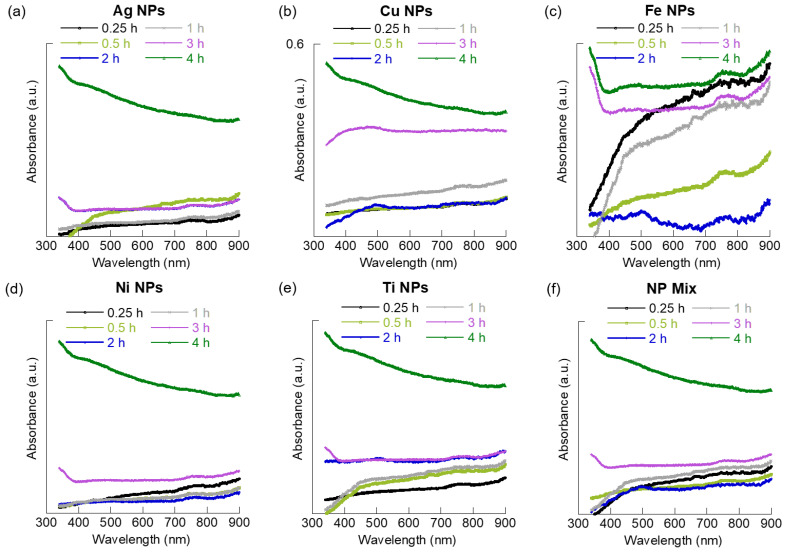
UV-Vis plots of the different nanoparticle samples at varying sonication times. (**a**) Ag, (**b**) Cu, (**c**) Fe, (**d**) Ni, (**e**) Ti, and (**f**) aqueous co-existed MNPs mix.

Individual MNPs: In general, dispersion of individual MNPs increased with increasing suspension time (Figure 1). For example, the dispersion of Ag and Ti NPs increased with the increasing suspension time, with the maximum occurring at 4 h. The relative quantities of Ag MNPs dispersed were similar for the 2 h and 3 h suspensions. In the case of Cu MNPs, the dispersion increased progressively with suspension time. However, a slight drop was observed at 2 h. For the Fe and Ni NPs, a 0.25 h suspension was found to disperse large quantities of NPs; however, the most dispersion was observed for suspension times of 3 and 4 h. Overall, the Cu, Ni, and Fe MNPs showed a similar trend in absorbance. The absorbance of these MNPs decreased after 2 h, compared to 0.25 h samples. We relate this to the change in the zeta potential of these MNPs with increasing sonication time. The surface charge of these MNPs showed significant change between 0.25 h and 2 h of sonication and can mechanistically account for the change in their dispersion at these sonication times. We attribute this change in zeta potential and absorbance profile to increased homo-aggregation in these MNPs. The absence of a defined absorbance peak at a specific wavelength for NPs, such as Ag and Cu, is due to the low concentration of MNPs used in our study to mimic environmentally relevant concentrations.

Co-existing MNPs: The relative amount of co-existing MNPs dispersed at 0.25 h was similar to the 1 h suspension and was lower at 0.5 h and 2 h with an increase observed at 3 h reaching maximum after a 4 h suspension (Figure 1f). Whereas the co-existing MNPs showed a bimodal distribution, similar to the dispersion of individual MNPs with suspension time, the co-existing MNPs showed decreased dispersion after 0.5 h suspension and after 2 h. We believe the decreased dispersion as early as 0.5 h is because of aggregation of the MNPs due to the increased number of particles that resulted in hetero-aggregation of NPs, compared to individual NPs dispersion where homo-aggregation is the only possibility. It is expected that the aggregates formed have a higher chance of settling to the bottom than non-aggregated NPs, even under sonication suspension conditions.

In general, the absorbance observations indicated that with increased time of suspension induced by mixing, within the time range evaluated, the MNPs dispersion in their original form may increase initially before they form aggregates. However, a further increase in the time of exposure may result in disaggregation. Decreased absorbance at intermediate times suggests the formation of aggregates and potential settling to the bottom. These results imply that when introduced into the aqueous environment, the turbulence in the water system may encourage MNPs initial dispersion, followed by aggregation. One could expect the aggregated MNPs to settle to the bottom of the water column if they have sufficient density and supportive flow parameters, such as quiescent conditions. However, continued turbulence on the unsettled aggregates will result in their re-dispersion in the water column.

### 3.2. Suspension Time versus MNPs Aggregation and Dispersion

For an increased understanding of the MNPs aggregation behavior as a function of suspension time (0.25, 2, and 4 h), three metrics are reported: the smallest hydrodynamic size showing a significant peak for the respective MNPs, the average hydrodynamic size, and the PdI. These measurements were based on intensity-weighted size measurements. The corresponding volume and number-based measurements for each MNP are provided in the Supplementary Materials.

**Figure 2:**
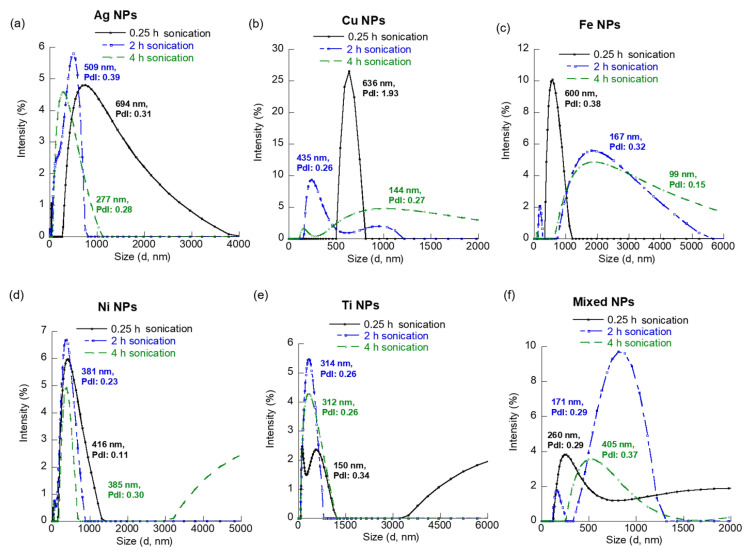
DLS size plots of the different NPs at various sonication times. (**a**) Ag NPs, (**b**) Cu NPs, (**c**) Fe NPs, (**d**) Ni NPs, (**e**) Ti NPs, and (**f**) co-existing MNP mix.

Individual MNPs: Ag NPs showed a significant peak at 694 nm with a PdI of 0.31 and an average hydrodynamic size of 557 nm after 0.25 h sonication (Figure 2a). However, smaller NPs (~40 nm) were also detected in this Ag NP sample. This multimodal size distribution of the Ag NPs indicated the presence of smaller NPs in the solution and their tendency to aggregate. Per the National Institute of Standards and Technology definition, an NP sample is considered mono-disperse for a PdI value up to 0.03, under the assumption that PdI is equal to σ/d (σ = standard deviation and d = mean NP size) for DLS measurements [24]. However, most commercial MNPs, such as the ones used in our study, show a higher polydispersity index [25,26]. Hence, we compare the relative variation in PdI with sonication time for our samples to understand the sonication-dependent changes in dispersion. The aggregation state was also visible in the Ag NPs sample after 2 h suspension, as the PdI slightly increased and the average hydrodynamic size showed a marked difference from the smallest detected size peak (Figure 2a, Table 2). However, significant improvement was observed in the dispersion and reduced aggregation of the Ag NPs after a 4 h suspension (Figure 2a). The PdI for this sample (0.28) indicated a single-size distribution and relatively uniform NP solution, which was further supported by a close match between the observed intensity-weighted peak (277 nm) and the hydrodynamic size (289 nm) of this sample. The Cu NPs showed heavy aggregation after a 0.25 h suspension, as suggested from the high PdI (1.93) and large average hydrodynamic size (1317 nm). The smallest size of NP aggregates in this sample was 636 nm (Figure 2b). The dispersion of this sample greatly improved after 2 h sonication with a PdI < 0.3 and an average hydrodynamic size of 435 nm. Some 144 nm Cu NPs were observed after longer suspension (4 h). However, the aggregation state, as well as the PdI, did not show further improvement, compared to the 2 h suspension.

For Fe NPs, the PdI progressively improved with sonication time. The smallest NP sizes observed decreased from 600 nm at 0.25 h suspension to 167 nm and 99 nm, at 2 h and 4 h suspension, respectively (Figure 2c). However, a bimodal distribution of NPs is observed after 2 h of sonication, indicating the presence of homo-aggregation. The Ni NPs showed the lowest PdI (0.11) and minimum aggregation (average hydrodynamic size = 338 nm) after 0.25 h suspension (Figure 2d). The dispersion of Ti NPs increased with suspension time, but no significant change was observed in the PdI and hydrodynamic sizes between the 2 h and 4 h sonicated samples (Figure 2e). The Ti NPs in aqueous phase showed a multimodal size distribution with the smallest NPs of size 150 nm, a PdI of 0.34, and an average hydrodynamic size of 458 nm after a 0.25 h suspension. The single size distribution was observed (PdI: 0.26 and average hydrodynamic diameter = 293 nm) after suspension of the Ti NP sample for 2 h.

**Figure 3:**
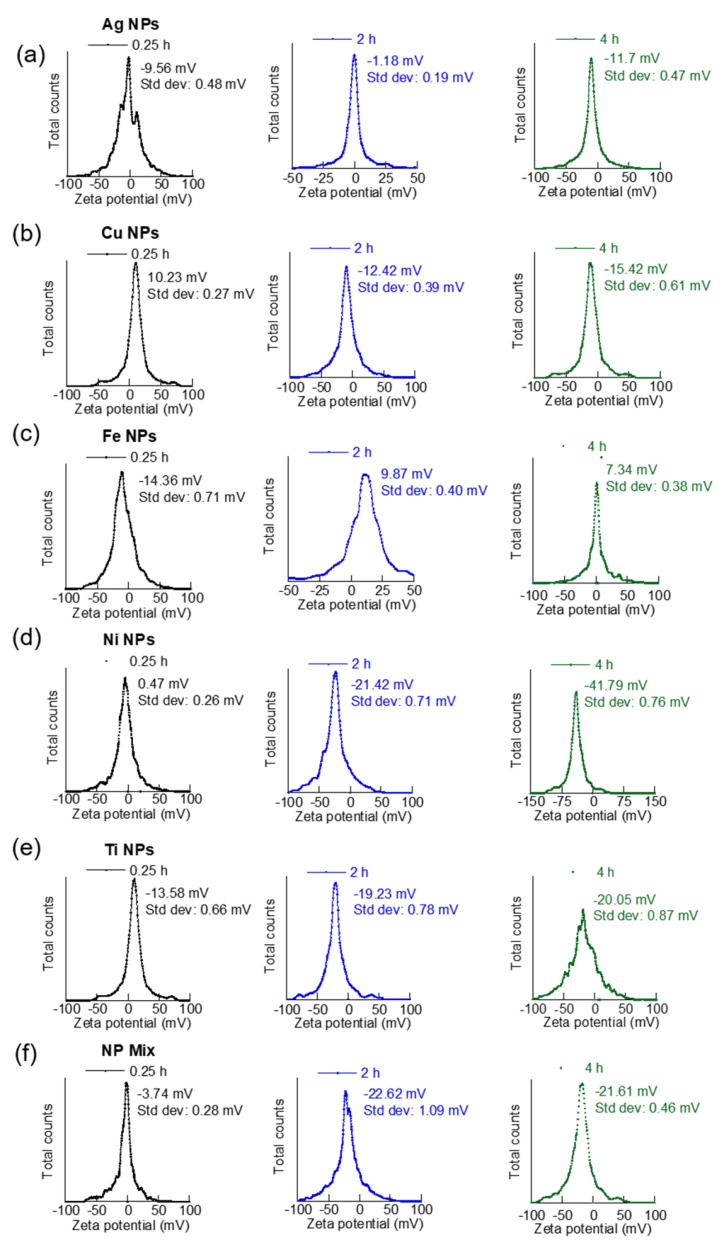
Zeta potential plots of different NPs at 0.25, 2, and 4 h of suspension. (**a**) Ag NPs, (**b**) Cu NPs, (**c**) Fe NPs, (**d**) Ni NPs, (**e**) Ti NPs, and (**f**) Co-existing MNP mix.

**Figure 4:**
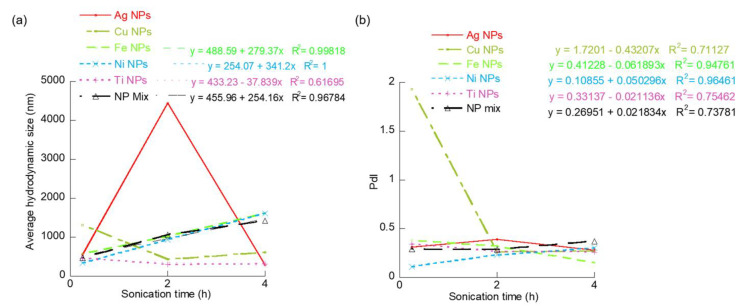
Effect of suspension time on dispersion quality of various MNPs in terms of (**a**) average hydrodynamic size and (**b**) PdI.

Suspension time versus zeta potential results showed a close correlation with our findings on nanoparticle dispersibility from their hydrodynamic sizes (Figure 3 and Figure 4). For example, the zeta potential of Ag NPs varied from −9.56 mV after 0.25 h sonication to -1.18 mV after a 2 h suspension and −11.7 mV after 4 h suspension. The broad peak for Ag NPs samples suspended for 0.25 h indicated the presence of heavy aggregation. In general, a higher absolute value for zeta potential reflects the higher stability of the aqueous NPs. The highest absolute value of zeta potential was observed at 4 h for these NPs, suggesting best dispersibility or least aggregation at these conditions (Figure 3a). Cu NPs suspended for 0.25 h showed a zeta potential of 10.23 mV. However, the zeta potential changed to –12.42 mV and −15.42 mV after a 2 h and 4 h suspension, respectively (Figure 3b). This was likely due to the dispersibility of more Cu NPs from disaggregation of earlier aggregates with increased suspension. The higher absolute value of zeta potential with increased sonication time also indicated an increased dispersion of NPs with prolonged suspension.

For Ni NPs, the absolute value of zeta potential progressively increased from 0.47 mV at 0.25 h to −21.42 mV and −41.79 mV at 2 h and 4 h, respectively (Figure 3d). The low stability of Ni NPs at 0.25 h could be due to the large amount of Ni NPs dispersing quickly in the aqueous medium. The stability of NPs increased with suspension time with highest stability seen from the highest absolute value of zeta potential at 4 h suspension. The dispersion quality of Ti NPs in the aqueous phase also improved with suspension time, which was reflected in the higher absolute value of zeta potential at 2 h (−19.23 mV), as compared to 0.25 h (−13.58 mV) (Figure 3e). However, there was a smaller change in zeta potential between 2 h and 4 h suspension for this sample.

Co-existing MNPs: The highest dispersion or lowest aggregation state for the co-existed sample containing all five MNPs was observed after a 0.25 h suspension. The average hydrodynamic size of this co-existing MNP sample was slightly higher (466 nm) than the lowest MNP sizes (260 nm) observed in the sample, suggesting the presence of slight aggregation. The PdI value was 0.29 for this sample (Figure 2f). Unlike, individual MNPs, the co-existing MNP increased hydrodynamic aggregation with suspension time due to hetero-aggregation facilitated by particle collision from their Brownian motion. However, higher suspension times did not increase the polydispersity of the co-existing MNPs, though some smaller sizes of MNPs (171 nm) were visible in the intensity-weighted size measurements of these samples. The absolute value of zeta potential of the co-existing MNP showed a significant increase from 3.74 mV at 0.25 h suspension to 22.62 mV after 2 h suspension with a slight decrease observed at 4 h (Figure 3f). This showed that the co-existed MNP was most stable at 2 h.

In general, the overall average hydrodynamic diameter of the MNPs increased with suspension time up to 2 h and decreased as the suspension time extended to 4 h (Figure 4). This observation is similar to the UV-Vis absorbance results noted in the study. The initial increase is believed to be the formation of aggregates caused by the Brownian motion of the MNPs. The extended sonication is believed to cause the disaggregation of the weak aggregates (or agglomerates). In fact, this increasing trend in average hydrodynamic size could be predicted through a forced linear fit (R^2^ > 0.97) for the Fe, Ni, and co-existing MNPs. The Ti NPs showed a decreasing trend in size with respect to suspension time that could be modeled by a forced linear equation with a regression fit (R^2^) of 0.62. In comparison, the PdI of the co-existing MNPs shows a slight decrease or remains similar with increased suspension time. We also observed that this variation in PdI with suspension time could be modeled by linear trendlines for Cu, Fe, Ni, Ti, and co-existing MNP samples (Figure 4b). One key conclusion from our DLS size analysis is the confirmation that the suspension time of MNPs in aquatic system plays a major role on the dispersion quality or in turn aggregation of the MNPs. However, as previously noted, the suspension time and extent of turbulent mixing are dependent on the river’s hydraulic and geomorphological conditions. Another major finding is that this effect on MNPs dispersibility, and hence their aggregation, is dependent on the type of MNP. The developed linear equations showing the variation of average hydrodynamic size and polydispersity index of the different MNP samples with suspension time can be easily used for preliminary prediction of the effects of MNPs suspension time on the overall size and PdI of five individual MNPs and their co-existed MNPs mix which determines their environmental fate.

### 3.3. Sonication Time versus Dispersion Concentration

**Figure 5:**
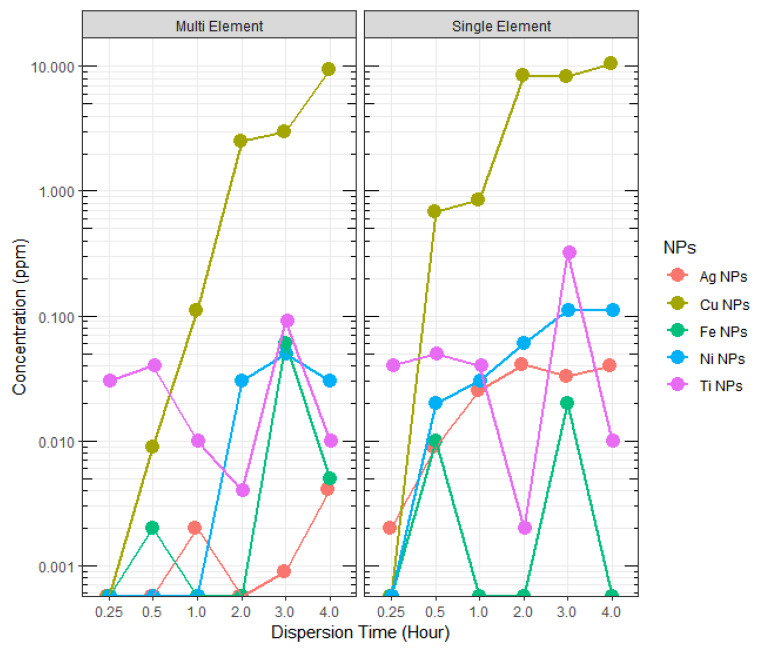
ICP-AES detected concentrations of MNPs in dispersion solutions at various dispersion times.

Individual MNPs: In general, higher concentrations of MNP were detected for longer suspension times (Table 3 and Figure 5), which is believed to be due to increased uniformity in MNPs dispersion. It is believed that the increased Brownian motion from continued sonication led to disaggregation of the weakly bonded MNP aggregates, resulting in their dispersion in the solutions. Both the ICP-AES and UV-Vis analyses showed that among the other MNPs, the Ni NPs achieved higher dispersion in an aqueous medium within 0.25 h of sonication. Moreover, the low or no detection at the beginning of the sonication, i.e., 0.25 h, may be caused by increased hydrophilic growth of MNPs once they are introduced into the aqueous media. However, the detected MNPs concentration did not always increase with the increase in suspension times. We suspect that the MNPs in suspension underwent aggregation initially, which upon continued suspension resulted in disaggregation.

The limitation of dispersion is directly evident in the calculated recoveries found in Table 4. The percent recoveries of Fe, Ni, Ag, and Ti NPs are less than 5% for the individual solutions. Of note are the very high Cu NPs recoveries, greater than 100%, which we suspect is due to sample contamination even after following analytical protocols. Such disparity in the maximum possible concentration versus the detected indicates the limitation of the bath sonication for dispersing the MNPs in aqueous media. In addition, one could conclude that when MNPs enter the aquatic environment they may aggregate if the turbulence in the environment is not sufficient to break the agglomerates to disperse the MNPs. The settling fate of such aggregates is dependent upon their density.

**Figure 6:**
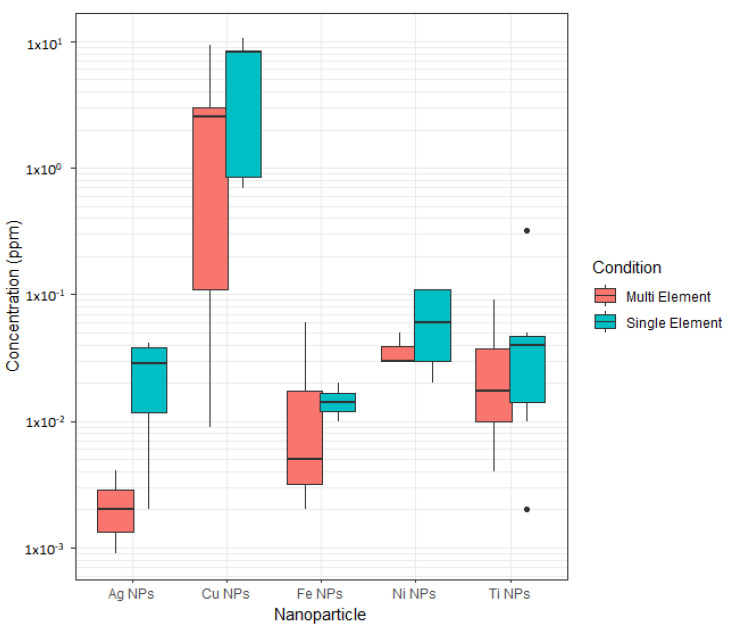
Comparison of ICP-AES detected concentrations of MNPs in individual element versus co-existing NP solutions.

Coexisting MNPs: In general, Ag, Cu, Ni, and Ti NPs were detected at the same or relatively higher concentrations in individual element samples, in comparison to the co-existing sample at all sonication times (with few exceptions) (Figure 6, Table 5). This observation can be attributed to the presence of a higher number of MNPs particles in coexisting solution increased the probability of particle collision frequency, which leads to the hetero-aggregation of particles and, hence limits their dispersed concentration [11]. Moreover, the use of 0.22 µm filter to draw the sub-sample for ICP-AES analysis would remove any aggregated particle sizes that are greater than 220 nm, which we believe is highly likely in the case of multi-element solutions. However, such an approach of filtration for ICP-AES analysis has been adapted from previous similar research [17]. As discussed earlier, DLS analysis of dispersion particle sizes showed an increase in MNPs hydrodynamic diameter above 200 nm in the samples. Such large (>200 nm) particle aggregates are outside the range of MNPs size and are close to a colloidal particle size, which may eventually settle in the bottom of a waterbody. Based on the detected concentrations (Figure 6), Ag NPs apparently underwent higher hetero-aggregation, compared to other NPs tested. Also, for Ti NPs, showed outliers on both sides of the box plot, and the distribution is negatively skewed (Figure 6), indicating the detected concentrations are not uniformly distributed around their mean. Overall, the aggregation behavior of individual NPs with others could not be concluded in this study.

In the real world, such high concentrations used in the coexisting MNPs samples for this study are expected only in the wastewater streams or receiving waters immediately downstream of wastewater discharge points or when a concentrated MNPs stock accidentally spills into receiving water. Hence, an important fate of the MNPs close to their discharge point in receiving waters could be an aggregation. Moreover, it is important to note that such aggregation behavior also depends on the chemistry of the media [27].

## 4. Conclusions

Here, three analytical techniques (UV-Vis, DLS, and ICP-AES) were used in tandem to gain key insights into the dispersion quality and aggregation state of the MNPs at different suspension times in aqueous samples. Although the increased suspension times enhanced the dispersion of the MNPs initially, the continued suspension caused greater Brownian motion and more aggregation, resulting in multimodal distribution dispersion of the MNPs. In general, the dispersion of the MNPs increased initially over the sonication times 0.25 h to 2 h but decreased between 2 h and 4 h. Dominant hetero-aggregation among MNPs in the co-existed samples has apparently favored aggregation of the MNPs, and hence their reduced dispersion, compared to individual MNPs samples. For the expected trace levels of the MNPs in a river environment, the hetero-aggregation may not be feasible among the MNPs. However, such aggregation may be possible with other ions present in the environmental water. Moreover, a higher concentration of the MNPs with potential for hetero-aggregation may be present in municipal and industrial wastewater streams. Despite the nominal size of the MNPs, they all appear to form large aggregates in water, in all the conditions explored.

The dispersion quality and hence aggregation and eventual fate of MNPs are dependent on their physicochemical properties. For example, because of their magnetic interactions, Fe NPs in the dispersion had different dispersion quality, compared to the rest of the MNPs tested. Moreover, some metal ions, such as Fe, are more soluble in the aqueous phase, compared to others (e.g., Ag, Cu, Ni, and Ti), which can also explain the difference in dispersion observed between the individual MNP systems. However, there are several other factors, such as hydrogen bonding, that can play a fundamental role in the dispersion of MNPs. The extended DLVO theory (X-DLVO) further advances the model by taking into account the short-range interactions beyond electrostatic and Van der Waals, as well as non-DLVO interactions (e.g., chemical bonding and hydration forces) [28]. Hydrogen bonding is a common interaction for metal MNPs in the aqueous phase, which is better explained through the X-DLVO model. The time-dependent dispersion behavior of the individual MNPs and co-existing MNPs can also be attributed to the formation of hydrogen bonds between the MNP and water molecules with prolonged sonication.

Further studies are warranted with real environmental samples to understand the role of co-existing MNPs and water chemistry impacting the aggregation and dispersion fate of the MNPs. Such studies will assist in the effective control of emerging MNPs pollution in aquatic systems. In addition to initiating an idea to depict environmental mixing conditions for the MNPs using bath sonication, this demonstrated use of the tandem techniques method to characterize MNPs has provided evidence on the application for environmental sample analysis.

## Figures and Tables

**Table 1:** Properties and dispersion sample concentrations of MNPs.

NP	Particle Diameter (nm)	Surface Area (m^2^/g)	True Density (g/cm^3^)	Purity (Trace Metal Basis)	MNPs Concentration in Single Element Solutions (mg/L)
Ag	20–30	20	10.5	99.95%	2
Cu	60–80	6–8	8.9	99.90%	2
Fe	40–60	6–13	7.9	99.70%	4
Ni	40–60	10–15	8.9	99.70%	6
Ti	40–60	20	4.5	99.90%	2

**Table 2:** DLS measured characteristics of MNPs in the dispersion solution.

NP	Sonication Time (Hours)	Average Diameter (nm)	PdI	ζ-Potential (mV)	U (μmcm/(Vs))
Ag	0.25	557.7	0.31	−9.56	−0.6775
	2	4440.3	0.39	−1.18	−0.0833
	4	289.4	0.29	−11.68	−0.8279
Cu	0.25	1317	1.93	10.22	0.7246
	2	435.5	0.27	−12.42	−0.8798
	4	615.2	0.27	−15.42	−1.0929
Fe	0.25	572.2	0.39	−14.36	−1.0178
	2	1021.5	0.32	9.87	0.6994
	4	1618.1	0.15	7.34	0.5200
Ni	0.25	338.5	0.11	0.47	0.0331
	2	938.1	0.23	−21.42	−1.5177
	4	1618.1	0.15	−41.79	−2.8716
Ti	0.25	458.2	0.34	−13.85	−0.9817
	2	293.0	0.26	−19.23	−1.3624
	4	312.0	0.26	−20.05	−1.4208
Co-existing MNP	0.25	466.0	0.29	−3.74	−0.2652
	2	1064.6	0.29	−22.62	−1.6028
	4	1425.8	0.37	−21.61	−1.5312

**Table 3:** Detected concentration of single element solutions MNPs.

Dispersion Time (Hours)	Ag NPs (ppm)	Cu NPs (ppm)	Fe NPs (ppm)	Ni NPs (ppm)	Ti NPs (ppm)
0.25	0.002	ND *	ND *	ND *	0.04
0.50	0.009	0.69	0.01	0.02	0.05
1.00	0.025	0.84	ND *	0.03	0.04
2.00	0.041	8.52	ND *	0.06	0.002
3.00	0.033	8.31	0.02	0.11	0.32
4.00	0.040	10.5	ND *	0.11	0.01
Actual Concentration in Prepared Solution (ppm)	2	2	4	6	2

* ND: not detected above the LOQ.

**Table 4:** Calculated recovery (%) of individual MNPs in different sonication times.

Dispersion Time	0.25 h	0.5 h	1 h	2 h	3 h	4 h
MNP						
Fe	0	0.250	0	0	0.500	0
Ni	0	0.333	0.500	1.00	1.83	1.83
Ag	0.100	0.450	1.25	2.05	1.65	2.00
Ti	2.00	2.50	2.015	0.100	16.0	0.500
Cu *	0 *	34.5 *	42.0 *	426 *	415 *	525 *

* Values are suspect.

**Table 5:** Detected concentration of MNPs in co-existing solutions.

Dispersion Time (h)	Ag NPs (ppm)	Cu NPs (ppm)	Fe NPs (ppm)	Ni NPs (ppm)	Ti NPs (ppm)
0.25	ND *	ND *	ND *	ND *	0.03
0.50	ND *	0.009	0.002	ND *	0.04
1.00	0.002	0.11	ND *	ND *	0.01
2.00	ND *	2.53	ND *	0.03	0.004
3.00	0.0009	3.01	0.06	0.05	0.09
4.00	0.0041	9.41	0.005	0.03	0.01
Actual Concentration in Prepared Solution (ppm)	12	12	12	12	12

* ND: not detected above the LOQ.

## Supplementary Materials

The following supporting information can be downloaded at: https://www.mdpi.com/article/10.3390/ma15196733/s1, Figure S1: Flowchart depicting experimental protocol of prepared and analyzed NP dispersions, Figure S2. Volume-based DLS size plots of the Nanoparticles at different sonication times, Figure S3: Number-based DLS size plots of the Nanoparticles at different sonication times, Table S1: ICP-AES Operating Conditions, Table S2: Emission wavelength, LOD, and LOQ.
