# Supplementary material for: Dispersion and Aggregation Fate of Individual and Co-Existing Metal Nanoparticles under Environmental Aqueous Suspension Conditions
Source: Materials (Basel). 2022 Sep 28;15(19):6733. doi: 10.3390/ma15196733 (PMC9572943; doi:10.3390/ma15196733)
Supplement: Supplementary file 1 [file materials-15-06733-s001.zip › materials-1903886-supplementary.pdf]

Supplementary Materials

Dispersion and Aggregation Fate of Individual and Co-existing Metal Nanoparticles under Environmental Aqueous Suspension Conditions

The flow chart of five individual metal nanoparticles and their mix (i.e., co-existing) in the distilled water dispersion preparation and analysis is illustrated in Figure S1. Variable sonication times used are 0.15 hr, 0.5hr, 1 hr, 2 hr, and 5 hr.

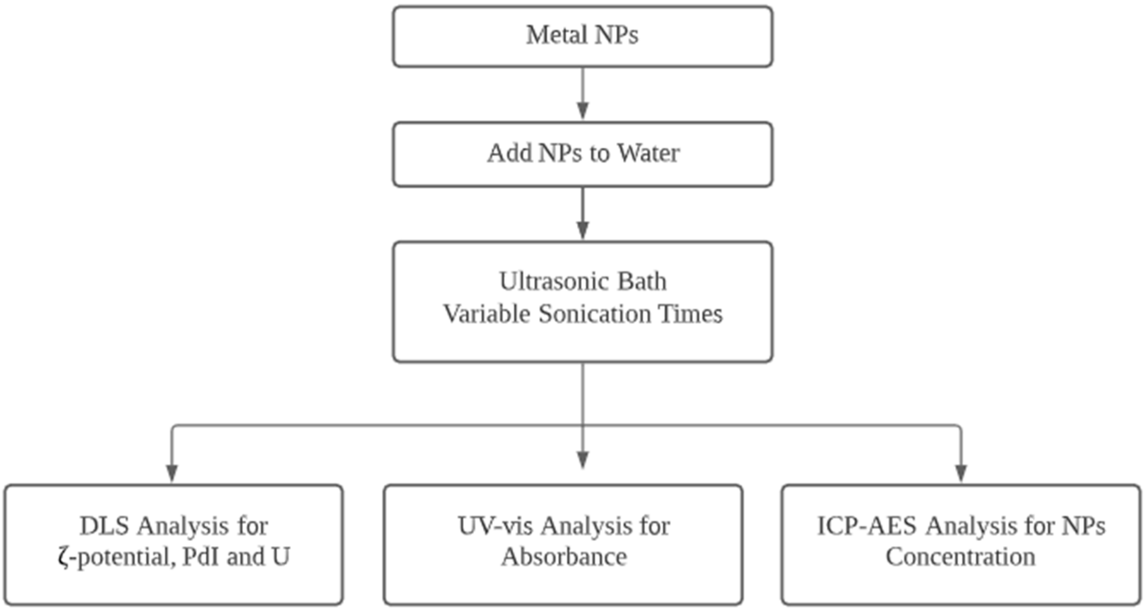

Figure S1. Flowchart depicting experimental protocol of prepared and analyzed NP dispersions

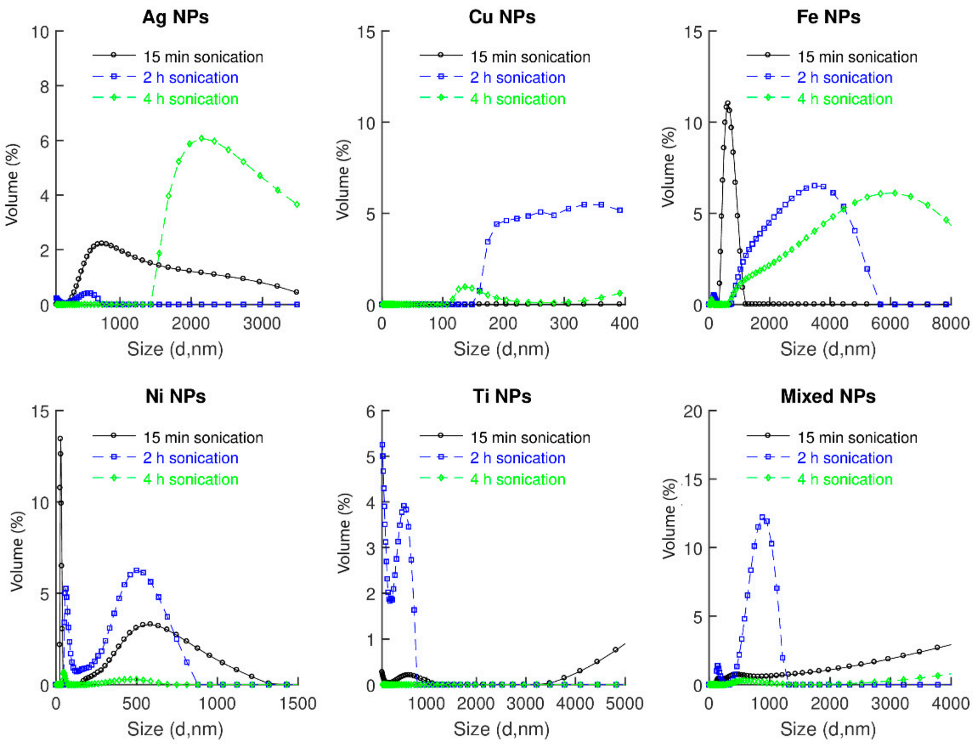

Figure S2. Volume-based DLS size plots of the Nanoparticles at different sonication times.

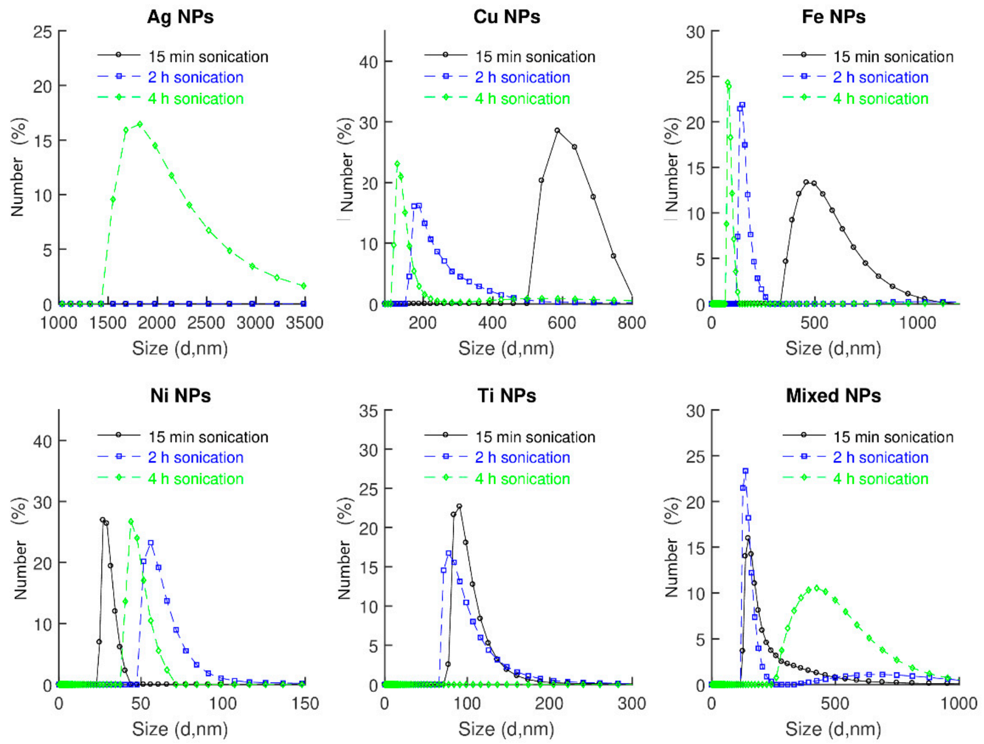

Figure S3. Number-based DLS size plots of the Nanoparticles at different sonication times.

The operating conditions of the inductively coupled plasma atomic emission spectroscopy (ICP-AES) are provided in Table S1. Table S2 contains the elemental analysis wavelengths, calibration equations, correlation coefficients, calculated limit of detection (LOD), and limit of quantitation (LOQ). All five of the calibration curves have a 10^4 linear dynamic range with excellent fit, indicated by correlation coefficients greater than 0.999.

Table S1: ICP-AES Operating Conditions

Radio Frequency	1.5 kW at 40.86 MHz

Power	1150 W
Plasma gas flow rate	15 L/min
Pump rate	1.0 mL/min
Monochromator grating	Czery-Turner, 1.0 m, 2400 grooves/mm, holographic
Entrance slit width	20 nm
Exit slit width	80 nm

Table S2: Emission wavelength, LOD, and LOQ

Element	Wavelength (nm)	Calibration Equation	Correlation Coefficient	LOD (ppm)	LOQ (ppm)
Ag	328.068	$y=1.25 \times 10^6x$	0.9997	0.000726	0.00242
Cu	324.754	$y=1.14 \times 10^6x$	0.9994	0.000473	0.00158
Fe	259.940	$y=1.36 \times 10^6x$	0.9999	0.000503	0.00168
Ni	231.064	$y=9.45 \times 10^6x$	0.9997	0.00231	0.00771
Ti	334.941	$y=1.08 \times 10^6x$	0.9993	0.000472	0.00157
